# Leveraging Hamiltonian neural flow for robust single-cell multi-omics integration: application to Alzheimer’s disease

**DOI:** 10.3389/fgene.2026.1795752

**Published:** 2026-04-24

**Authors:** Ziheng Huang, Wei Kong, Shuaiqun Wang

**Affiliations:** College of Information Engineering, Shanghai Maritime University, Shanghai, China

**Keywords:** Alzheimer’s disease, graph neural networks, Hamiltonian dynamics, interpretable machine learning, single-cell multi-omics

## Abstract

Alzheimer’s disease (AD) progression involves complex molecular interactions across multiple biological layers, yet integrating high-dimensional single-cell multi-omics data remains computationally challenging. While Graph Convolutional Networks (GCNs) effectively model cell-gene interaction topologies, they face three critical limitations: over-smoothing in deep architectures, instability under data perturbations, and lack of mechanistic interpretability—obstacles that impede clinical translation. The Hamiltonian Graph Convolutional Network (HGCN), a physics-inspired framework integrating symplectic dynamics with graph-based learning, is proposed in this study, which incorporates energy-conserving Hamiltonian mechanics to address these limitations through: (1) geometric constraints that prevent over-smoothing, (2) stable gradient propagation via symplectic integration, and (3) interpretable phase space representations of cellular states. To validate the effectiveness of the HGCN model, it was evaluated on three single-cell multi-omics datasets: an AD prefrontal cortex dataset, and peripheral blood benchmarks. Meanwhile, differential analysis emerged as the most effective feature extraction strategy in the evaluated experimental setting through systematic preprocessing comparisons. On the AD composite classification task requiring simultaneous prediction of cell type and disease state, HGCN achieved 92.28% accuracy and 0.9228 F1-score, significantly outperforming baseline GCN (88.59% accuracy, 0.8860 F1-score). Phase space visualization revealed biologically meaningful patterns: Inhibitory neurons exhibited heterogeneous subtype structures, while disease states showed symmetric geometric organization suggesting cell-type-invariant pathological mechanisms. Robustness experiments on citation networks demonstrated superior resilience to both feature and structural perturbations compared to standard GCN, with performance advantages increasing under higher perturbation intensities. These results establish HGCN as a robust, interpretable framework for multi-omics integration in complex disease analysis, with potential applications in precision medicine.

## 1 Introduction

Alzheimer’s disease (AD) represents one of the most complex neurodegenerative disorders, with pathogenesis driven by intricate interactions among genetic, epigenetic, transcriptomic, and environmental factors (Masters et al., 2015; De Strooper and Karran, 2016). Single-omics approaches, while providing valuable insights, cannot capture the full molecular complexity underlying AD progression. At single-cell resolution, the joint profiling of transcriptomics (RNA-seq), epigenomics (ATAC-seq), and proteomics has emerged as essential for dissecting cellular heterogeneity, reconstructing regulatory networks, and identifying disease-relevant molecular signatures (Liu et al., 2019; Wang Y. et al., 2021). However, integrating these high-dimensional, heterogeneous data modalities remains a fundamental computational challenge (Ma A. et al., 2020).

Graph Neural Networks (GNNs), particularly Graph Convolutional Networks (GCNs), have demonstrated strong capabilities in modeling the non-Euclidean topologies inherent in biological networks (Kipf and Welling, 2017; Zhang et al., 2021). By representing cells as nodes and their relationships as edges, GCNs aggregate neighborhood information to learn informative embeddings that capture local graph structure. This framework naturally accommodates multi-omics integration: transcriptomic and epigenomic features can be encoded as node attributes, while cell-cell similarities or gene-gene interactions define the graph topology. Several recent studies have successfully applied GCNs to single-cell classification tasks, achieving competitive performance on benchmark datasets (Wang et al., 2021b; Song et al., 2021). Despite their promise, existing GCN-based methods face three critical limitations that hinder their application to complex biological systems.

First, as network depth increases (Li et al., 2018; Oono and Suzuki, 2020; Chen et al., 2020), node representations become increasingly similar through repeated neighborhood aggregation, causing embeddings to converge to indistinguishable vectors. This phenomenon, known as over-smoothing, severely degrades classification performance and limits the ability to model complex multi-scale biological processes that require deeper architectures. Second, GCNs exhibit high sensitivity to both feature noise and structural perturbations (Zhu et al., 2019; Zhang and Zitnik, 2020). Single-cell multi-omics data inherently contains technical noise from dropout events, batch effects, and measurement variability. Moreover, constructed cell-cell graphs may contain spurious edges or miss biologically relevant connections. Without explicit stability constraints, conventional GCNs propagate these errors through the network, leading to unreliable predictions that compromise clinical utility. Third, standard GCNs function as black-box models where the decision-making process remains opaque (Ying et al., 2019; Yuan et al., 2022). In biomedical applications, particularly for disease diagnosis and biomarker discovery, understanding why a model makes specific predictions is as crucial as predictive accuracy itself. The absence of interpretable representations limits biological insight generation and hinders clinical translation, where mechanistic understanding is essential for regulatory approval and physician trust.

Recent efforts have addressed isolated aspects of these challenges. For transcriptomic data, sigGCN incorporated gene-gene interaction networks to improve performance on scRNA-seq datasets, but remains limited to single modalities and still suffers from over-smoothing. Multi-omics fusion strategies such as simple concatenation of RNA-seq and ATAC-seq features have shown only marginal improvements (Wang et al., 2014; Leng et al., 2022), particularly in high-complexity datasets like AD neurons where tissue-specific regulatory signals are difficult to capture. In terms of robustness, some methods have been developed to resist graph structure perturbations but fail to address node-level perturbations, while others ensure robustness against feature noise but cannot defend against topological attacks. For multi-omics integration specifically, MOGONET fuses heterogeneous data for patient classification but lacks interpretability (Wang et al., 2021c), limiting its utility for biomarker identification. ExpFiGCN enhances interpretability through frequency-domain analysis (Wei et al., 2024), but its reliance on pre-defined similarity measures and lack of conceptual constraints restricts generalization. Critically, existing approaches fail to provide a unified framework that simultaneously ensures robustness, interpretability, and effective multi-omics integration while mitigating over-smoothing. This gap motivates the development of a theoretically grounded approach that addresses these fundamental limitations through principled mathematical constraints rather than empirical fixes.

To address these fundamental limitations, the Hamiltonian Graph Convolutional Network (HGCN), a novel framework that integrates principles from Hamiltonian mechanics into graph-based deep learning, is proposed. The framework combines rigorous multi-omics preprocessing with physics-inspired graph neural architecture to enable robust and interpretable disease analysis, which can provide four interconnected innovations. First, by imposing energy-conserving propagation mechanisms through Hamiltonian dynamics, geometric constraints are introduced that naturally prevent over-smoothing while preserving discriminative information across deep architectures. Unlike *ad hoc* regularization techniques, these constraints emerge from fundamental physical principles governing dynamical systems. Second, symplectic integration schemes are employed that maintain the fundamental geometric structure of phase space, ensuring stable gradient flow and enhancing robustness to both feature and structural perturbations. This approach provides theoretical guarantees on numerical stability that conventional integration methods cannot offer. Third, node embeddings are decomposed into position q and momentum p coordinates, creating a phase space where cellular states evolve along energy-preserving trajectories. This formulation provides mechanistic interpretability: visualizations reveal how cells transition between states and how disease effects manifest as geometric transformations in phase space. Fourth, the GCN component efficiently aggregates multi-scale features from transcriptomic and epigenomic data, while Hamiltonian dynamics reduce computational complexity compared to pure ODE-based approaches through fixed-step symplectic integration.

The primary goal of this work is to model Alzheimer’s disease–related cellular state dynamics using a physics-inspired framework. Nevertheless, because publicly available AD single-cell multi-omics datasets remain extremely limited, our experimental evaluation consists of one AD brain multi-omics dataset as the central disease application, together with widely used peripheral blood benchmarks for auxiliary validation: an AD prefrontal cortex dataset (GSE214979, 96,000+ cells with paired RNA-seq and ATAC-seq across 8 cell types and 2 disease states), and peripheral blood benchmarks (PBMC-10k with 9,631 cells and 19 immune subtypes; Ma-2020 with 5,692 cells and 22 immune populations). On the AD composite classification task requiring simultaneous prediction of cell type and disease state, HGCN achieved 92.28% accuracy and 0.9228 F1-score, substantially outperforming baseline GCN (88.59% accuracy, 0.8860 F1-score). Phase space visualizations revealed biologically meaningful patterns: Inhibitory neurons exhibited fragmented, thread-like structures reflecting known transcriptomic heterogeneity, while disease states showed symmetric geometric organization suggesting cell-type-invariant disease effects. Robustness experiments on citation network benchmarks (CiteSeer, PubMed) demonstrated superior resilience to perturbations, with performance advantages increasing as perturbation intensity grew.

## 2 Methods

### 2.1 HGCN model flowchart

**FIGURE 1:**
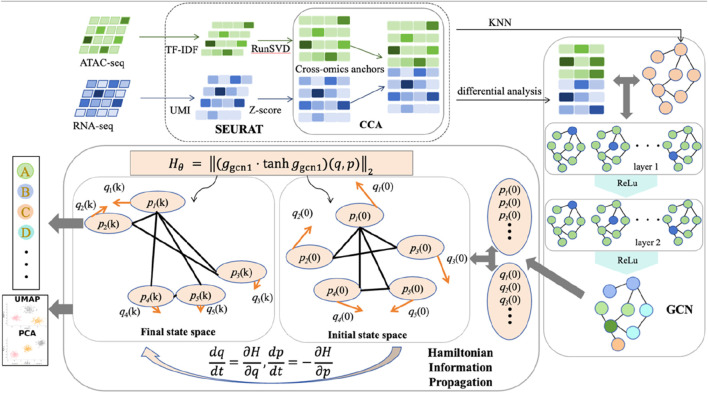
HGCN model flowchart.

As shown in Figure 1, it illustrates the complete workflow of HGCN. The HGCN framework was designed to address the fundamental limitations of conventional GCNs through a systematic integration of multi-omics data processing and Hamiltonian dynamics, which consists of three main components: (1) a standardized multi-omics preprocessing pipeline that integrates transcriptomic and epigenomic features; (2) a graph construction module that builds cell-cell similarity networks; and (3) the core HGCN architecture that combines graph convolution with Hamiltonian dynamics.

The proposed approach follows a systematic pipeline integrating data preprocessing, graph construction, and physics-inspired learning. A rigorous preprocessing workflow was implemented to standardize transcriptomic and epigenomic data, where differential analysis emerged as the most effective feature extraction strategy in the evaluated experimental setting through systematic preprocessing comparisons. Cell-cell similarity networks were then constructed using k-nearest neighbors on the integrated feature space based on weighted nearest neighbor distances. The core HGCN architecture decomposes learned representations into position q and momentum p coordinates, establishing a phase space where cellular states evolve along energy-preserving trajectories governed by Hamiltonian dynamics. Symplectic integration schemes were employed to maintain the fundamental geometric structure of phase space, ensuring stable gradient propagation and enhanced robustness to perturbations.

The HGCN architecture introduces a novel integration of Hamiltonian mechanics into graph-based learning. Unlike conventional neural networks that treat features as static vectors, HGCN establishes a phase space representation governed by energy conservation principles. This formulation provides three key advantages: (1) energy conservation constraints naturally prevent over-smoothing; (2) symplectic geometry ensures stable gradient propagation; and (3) phase space dynamics offer mechanistic interpretability of cellular state transitions.

### 2.2 Data preprocessing and feature extraction

#### 2.2.1 Datasets

Three categories of single-cell multi-omics datasets were utilized:

Alzheimer’s Disease Dataset (GSE214979) (Anderson et al., 2019): This dataset comprises 105,332 single-nucleus profiles from the prefrontal cortex of 7 AD patients and 8 healthy controls, with paired single-nucleus RNA-seq (snRNA-seq) and chromatin accessibility (snATAC-seq) measurements. After quality control, 96,427 high-quality cells spanning 8 cell types (Excitatory, Inhibitory, Oligodendrocytes, Astrocytes, OPCs, Microglia, Pericytes, Endothelial) and 2 disease states (AD, Control) were retained for analysis.

Peripheral Blood Benchmarks: The 10x Multiome PBMC dataset (10x Genomi cs, 2021) (9,631 cells) provides paired scRNA-seq and scATAC-seq profiles across 19 immune subtypes with a 29,095-dimensional chromatin accessibility matrix. The Ma-2020 dataset (Ma S. et al., 2020) contains 5,692 cells with 21,478-dimensional scATAC-seq features spanning 22 annotated immune populations.

#### 2.2.2 Multi-omics preprocessing pipeline

For the GSE214979 dataset, a rigorous preprocessing workflow was implemented: RNA-seq Processing: Raw count matrices were normalized using the LogNormalize method with a scale factor of 10,000 and natural logarithm transformation (log(1 + x)), followed by scaling to ensure zero mean and unit variance for each gene (Butler et al., 2018; Stuart et al., 2019). Highly variable genes were identified using variance-stabilizing transformation (vst method) with the top 2,000 features selected (Hafemeister and Satija, 2019) (selection.method = 'vst’, nfeatures = 2000). This approach reduces noise from low-expression genes while retaining biologically informative variation.

ATAC-seq Processing: Peak accessibility matrices were evaluated using nucleosome signal and transcription start site (TSS) enrichment scores for quality control. Cells with nucleosome signal >2 or TSS enrichment <2 were filtered out. Term frequency-inverse document frequency (TF-IDF) normalization was applied following the standard formula: TF-IDF = log(TF × IDF) (Cusanovich et al., 2015; Satpathy et al., 2019), where TF represents term frequency (peak accessibility in each cell) and IDF = log(1 + N/df), with N denoting the total number of cells and df representing document frequency (number of cells where the peak is accessible). Feature selection and dimensionality reduction were performed via Latent Semantic Indexing (LSI) using the top 50 components, followed by Singular Value Decomposition (SVD) (Granja et al., 2019) to capture the major axes of chromatin accessibility variation.

Multi-Modal Integration and Graph Construction: Given the transductive learning setting employed in this study, graph construction was performed once using all cells to leverage the complete data structure, which is standard practice in graph neural network research. The construction pipeline proceeded through three stages: first, modality-specific k-nearest neighbor graphs were computed with k = 20 neighbors for both RNA (based on Euclidean distance in the top 2,000 highly variable gene space) and ATAC (based on cosine similarity in the top 50 LSI component space). Second, Weighted Nearest Neighbor (WNN) integration was performed using the Seurat v4 WNN algorithm (Hao et al., 2021) with parameters k.nn = 20, k.score = 20, and compute.SNN = TRUE, which learns modality weights through cross-modality prediction accuracy via mutual nearest neighbors and produces a unified cell-cell similarity matrix combining both transcriptomic and epigenomic information. Third, a final consensus k-nearest neighbor graph was constructed from the WNN distance matrix using k = 15 neighbors, producing an undirected, unweighted graph stored in PyTorch Geometric edge_index format. The hyperparameters were selected based on established best practices from the single-cell genomics and graph neural network communities: k = 20 for modality-specific graphs follows Seurat WNN recommendations to capture sufficient local neighborhood structure while maintaining computational efficiency and is widely used in single-cell analysis pipelines; k = 15 for the final consensus graph was selected based on sigGCN and scGCN benchmarks showing that k ∈ (Li et al., 2018; Wei and Mei, 2024) provides optimal performance for GCN-based single-cell classification, balancing local neighborhood information with noise robustness; 50 LSI components follows Signac package defaults and ArchR recommendations for single-cell ATAC-seq analysis, with prior studies demonstrating that 30–100 components capture the majority of chromatin accessibility variance with diminishing returns beyond 50; and 2,000 highly variable genes is standard in Seurat v4 and widely adopted in scRNA-seq preprocessing to retain biologically informative variation while reducing computational burden. All graphs were constructed using fixed random seeds to ensure deterministic neighbor selection in tie-breaking scenarios and enable full reproducibility.

#### 2.2.3 Differential analysis for feature extraction

To identify the most informative features for classification while avoiding information leakage, differential analysis was performed exclusively on the training set, with the selected features then frozen and applied to validation and test sets.

Differential Expression Analysis (RNA): Wilcoxon rank-sum tests were used to identify genes with significantly different expression between AD and control groups, resulting in 4,211 differentially expressed genes.

Differential Accessibility Analysis (ATAC): Using the same statistical framework, chromatin accessibility analysis identified 1,983 differentially accessible peaks associated with disease status.

Comparison with Alternative Methods: To validate this approach, differential analysis was compared with label-agnostic methods,Variance-based selection (top N highly variable features): 79.82% accuracy, Mutual information-based selection: 46.66% accuracy, Recursive feature elimination (RFE): 46.46% accuracy.

Training-set-only differential analysis achieved 93.57% accuracy, confirming its empirical advantage in this dataset and setting while maintaining proper train-test separation. For cell type classification, ATAC-derived features showed approximately 20% higher accuracy than RNA features, while both modalities contributed equally to disease state classification.

### 2.3 Graph convolutional network foundation

Given a set of N cells, a graph $G=\left(V,\varepsilon \right)$ is constructed where nodes V represent cells and edges ε encode cell-cell similarities.The adjacency matrix $A\in R^{N\times N}$ is constructed using k-nearest neighbors (k = 15) based on either transcriptomic features, epigenomic features, or their weighted combination. For datasets lacking inherent graph structure, this k-NN approach effectively captures local cellular neighborhoods while maintaining computational tractability.

The input feature matrix $X\in R^{N\times F}$ contains F features for each of N cells. A single GCN layer performs the following transformation:

$$H^{\left(l+1\right)}=\sigma \left({{\tilde{D}}^{-\frac{1}{2}}\tilde{A}{\tilde{D}}^{-\frac{1}{2}}H^{\left(l\right)}W}^{\left(l\right)}\right)$$ (1)

where: $\tilde{A}=$ A + $I_{N}$ is the adjacency matrix with added self-loops, $\tilde{D}$ is the diagonal degree matrix of $\tilde{A}$ , ${\;W}^{\left(l\right)}\in R^{F\times F^{’}}$ is a learnable weight matrix, σ ( · ) is a non-linear activation function, such as ReLU, $H^{\left(l\right)}$ denotes the feature representation at layer l, with $H^{\left(0\right)}=X$ .

This formulation enables each node to aggregate information from its local neighborhood through normalized summation, followed by a learnable linear transformation and non-linearity.

While effective for shallow architectures, standard GCNs suffer from over-smoothing as depth increases. The repeated neighborhood averaging causes node features to become increasingly similar, eventually converging to a space where all nodes are indistinguishable. Mathematically, as the number of layers L→∞ , all node representations converge to the same stationary distribution determined by the graph structure alone, regardless of initial features. This fundamentally limits the expressiveness of deep GCN architectures and motivates our integration of Hamiltonian dynamics.

### 2.4 Hamiltonian graph convolutional network (HGCN)

#### 2.4.1 Hamiltonian mechanics: foundational principles

In classical mechanics, the state of a physical system is completely described by generalized coordinates: position q and momentum p (Goldstein et al., 2001; Arnold, 1989). These coordinates span a phase space where the system evolves according to Hamilton’s equations:

$$\frac{dq}{dt}=\frac{\partial H}{\partial q},\frac{dp}{dt}=-\frac{\partial H}{\partial p}$$ (2)

where $H\left(q,p\right)$ is the Hamiltonian function representing the total energy of system. A fundamental property of Hamiltonian systems is the conservation of energy: $\frac{\partial H}{\partial t}=$ 0 along system trajectories. This conservation law, combined with the symplectic structure of phase space, ensures that:

Volume Preservation (Liouville’s Theorem): Phase space volume is preserved under Hamiltonian flow.

Reversibility: Trajectories can be traced backward in time.

Stability: Small perturbations lead to bounded deviations rather than exponential divergence.

These properties make Hamiltonian dynamics ideal for learning stable, interpretable representations in neural networks.

#### 2.4.2 HGCN architecture

Phase Space Initialization: The HGCN architecture begins with a standard GCN layer that processes the input feature matrix $X\in R^{N\times F}$ :

$$Z=\text{ReLU}\left({\tilde{D}}^{-\frac{1}{2}}\tilde{A}{\tilde{D}}^{-\frac{1}{2}}XW\right)\in R^{N\times H}$$ (3)

where H is the hidden dimension. This learned representation is then linearly projected into a 2 H -dimensional phase space:

$$Y_{0}=\text{Linear}\left(Z\right)=\left[q_{0}∥p_{0}\right]\in R^{N\times 2H}$$ (4)

where ∥ denotes concatenation, $q_{0}\in R^{N\times H}$ represents initial positions, and $p_{0}{\in R}^{N\times H}$ represents initial momenta.Note that bold notation is adopted consistently throughout: bold uppercase letters ( X ***,*** Z ) denote matrices, while bold lowercase letters $\left(q,p\right)$ denote vectors or vector-valued functions.

Learnable Hamiltonian Function: The HGCN architecture employs a learnable Hamiltonian that is optimized during training. Specifically, the following formulation is implemented:

$$H_{\theta }=∥\left(g_{\text{gcn}1}\cdot \tanh\cdot g_{\text{gcn}2}\right)\left(q,p\right){∥}_{2}$$ (5)

where $g_{\text{gcn}1}$ and $g_{\text{gcn}2}$ are GCN layers with different hidden dimensions, θ represents all learnable parameters, and · denotes function composition. The tanh activation ensures bounded-input-bounded-output (BIBO) stability, while the ℓ_2_ norm computes the scalar energy magnitude.

Implementation Details: This learnable Hamiltonian (Equation 5) is the one actually used in all forward passes during training and inference. The network architecture is: Linear(2 h → 4 h) → ReLU → Linear(4 h → 1) → ℓ_2_ norm. Gradients are computed via automatic differentiation in PyTorch. The symplectic Euler integrator uses these gradients to update phase space coordinates.

#### 2.4.3 Hamiltonian design rationale and generalization

The learnable Hamiltonian formulation (Equation 5) is motivated by three key principles that align with the structure of single-cell multi-omics data:

Energy Landscape Metaphor:In statistical mechanics, cellular states can be conceptualized as occupying positions in an energy landscape, where stable cell types correspond to energy minima (attractors) and transitions between states involve crossing energy barriers (Greydanus et al., 2019; Amari, 2010). The Hamiltonian framework naturally captures this structure, with the learned energy function $H\left(q,p\right)$ defining a data-driven landscape where: Position q represents cell identity/state in gene expression space.Momentum p encodes regulatory dynamics and epigenetic potential - Energy wells correspond to discrete cell types. Saddle points represent transitional states.

Geometric Regularization:The symplectic integration constraint acts as an implicit regularizer that preserves volume in phase space (Liouville’s theorem). For single-cell data, this ensures that:Learned representations maintain meaningful distance relationships. Rare cell populations are not collapsed into dominant clusters. Perturbations (technical noise, dropout) cause bounded rather than catastrophic deviations. Energy conservation is a theoretical consequence of these geometric properties in continuous time, and while symplectic integrators approximately preserve energy, strict conservation is neither required nor expected in discrete numerical implementation. What matters for our application is the geometric structure that constrains how node representations evolve, not perfect energy conservation. This distinction is important: Hamiltonian formalism is used as a principled way to define dynamics with desirable geometric properties, not to simulate physical energy conservation.

**FIGURE 2:**
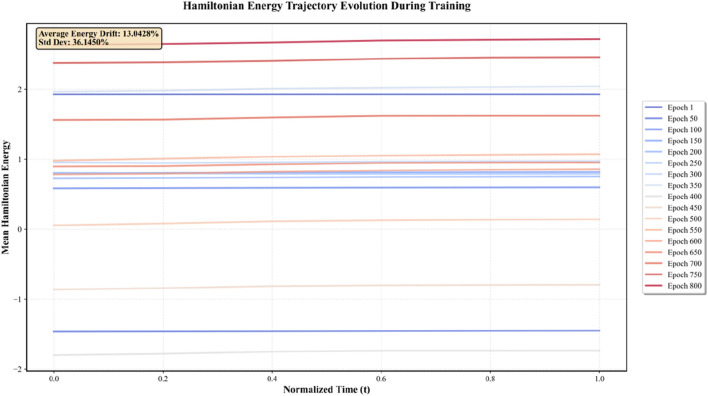
Energy trajectory evolution during training.

To empirically validate the energy-preserving properties of the symplectic integrator, Hamiltonian values were monitored throughout the training process on the GSE214979 dataset. Figure 2 shows the evolution of mean Hamiltonian energy across integration time steps for multiple training epochs.

The empirical analysis reveals: 1.Bounded Energy Fluctuation. The average energy drift across all training epochs is 13.04% with a standard deviation of 36.15%. While this indicates some fluctuation, the energy remains bounded within a finite range throughout training, confirming that the symplectic Euler integrator maintains approximate energy conservation at the numerical level. 2. Epoch-Dependent Stabilization. Energy trajectories show higher variance in early epochs (Epoch 1–200) and progressively stabilize in later epochs (Epoch 500–800), suggesting that as the model converges, the learned representations settle into more stable energy manifolds. 3. No Catastrophic Divergence. Critically, no exponential energy growth or collapse is observed, which would indicate numerical instability. The energy trajectories remain within approximately ±2 units of their initial values across normalized time, demonstrating that the discretization error is bounded and does not accumulate catastrophically.

### 2.5 Stability analysis

#### 2.5.1 Bounded-input-bounded-output (BIBO) stability

The learnable Hamiltonian function $H\left(q,p\right)$ exhibits BIBO stability due to the tanh activation and $l_{2}$ norm:

Theorem 1: (BIBO Stability): If ∥q∥ < ∞ and ∥p∥ < ∞ , then $H\left(q,p\right)$ < ∞ .

BIBO stability ensures that the system does not produce unbounded energy values during training, which would violate energy conservation principles and lead to numerical instability.

#### 2.5.2 Lyapunov stability via Lagrange-Dirichlet theorem

While BIBO stability guarantees bounded outputs, it does not ensure convergence to stable equilibria. To establish Lyapunov stability, the Lagrange–Dirichlet theorem from classical mechanics is invoked:

Theorem 2: (Lagrange-Dirichlet): An equilibrium point $\left(q^{*},p^{*}\right)$ of a Hamiltonian system is stable if:

1. Total energy is conserved: $\frac{\partial H}{\partial t}$ = 0
2. Potential energy $V\left(q\right)$ attains a strict local minimum at $q^{*}.$

Constructive Example for Stability Analysis:

To demonstrate that a Hamiltonian with the network architecture can satisfy these conditions, the following decomposition is provided:

1. Kinetic Energy:
$$T\left(q,p\right)=\sum_{k=1}^{N}p_{k}^{T}\left(A_{G}\left(q_{k}\right)A_{G}^{T}\left(q_{k}\right)+\sigma Ι\right)p_{k}$$ (6)
2. Potential Energy:
$$V\left(q\right)=∥\sin\left(q\right){∥}_{2}$$ (7)
  where $A_{G}$ is either the fixed adjacency matrix or a learnable attention matrix, and σ >0 is a small positive constant ensuring positive definiteness. Equations 6, 7 define a constructive example that satisfies the Lagrange-Dirichlet conditions, showing that a Hamiltonian with our structure can exhibit Lyapunov stability. However, the Hamiltonian actually learned (Equation 5) is not restricted to this exact form; the learned $H_{\theta }$ implicitly encodes the kinetic and potential energy components through its neural architecture.The choice of $V\left(q\right)$ = $∥\sin\left(q\right){∥}_{2}$ in the theoretical example introduces multiple local minima (at q = nπ for integer n), providing multiple stable equilibrium points. This multi-well potential enhances robustness: perturbations may shift the system to a nearby equilibrium rather than causing complete destabilization.

### 2.6 Training and hyperparameters

#### 2.6.1 Loss function

For node classification tasks, the cross-entropy loss is minimized over labeled nodes:

$$L=-\frac{1}{\left|V_{L}\right|}\sum_{i\in V_{L}}\sum_{c=1}^{C}y_{ic}\log{\hat{y}}_{ic}$$ (8)

where $V_{L}$ is the set of labeled nodes, C is the number of classes, $y_{ic}\in$ {0,1} is the ground-truth label, and ${\hat{y}}_{ic}$ is the predicted probability.

For the multi-task setting on GSE214979 (simultaneous cell type and disease state prediction), a weighted multi-task loss is employed: $L_{\text{total}}$ = *α* $L_{\text{type}}$ + *β* $L_{\text{state}}$ .

Where $L_{\text{type}}$ is the cell type classification loss, $L_{\text{state}}$ is the disease state classification loss, and *α*,*β* are balancing weights (set to 0.7 and 0.3 respectively based on validation performance).

#### 2.6.2 Hyperparameter configuration

Table 1 summarizes the key hyperparameters used across all experiments, all models (HGCN and baseline GCN) were trained using identical hyperparameters except for the Hamiltonian-specific components. Early stopping with patience of 50 epochs was employed based on validation loss.

**TABLE 1:** The configuration of hyperparameters.

Hyperparameter	Value	Description
Hidden dimension (*H*)	256	Dimension of GCN hidden layer
Phase space dimension	512 (2 × 256)	Total dimension of ( q,p )
k-nearest neighbors	15	Graph construction parameter
Integration time step (*Δt*)	0.2	Symplectic euler step size
Integration horizon	[0, 1]	Time interval for ODE solving
Learning rate	0.01	Adam optimizer learning rate
Training epochs	300–800	Dataset dependent
Dropout rate	0.5	Applied after GCN layer
Weight decay	5 × 10^−4^	L2 regularization

#### 2.6.3 Data splitting and evaluation protocol

To evaluate the donor-grouped splitting strategy, three classification tasks of increasing complexity were conducted on the GSE214979 dataset under identical random seeds: binary disease state classification (2 classes: AD vs. Control, HGCN: 88.26%, GCN: 72.94%), cell type classification (8 classes: major cell populations, HGCN: 98.19%, GCN: 97.62%), and composite classification (16 classes: 8 cell types × 2 disease states, HGCN: 87.26%, GCN: 71.18%). These results demonstrate that HGCN consistently outperforms GCN across all classification granularities, with performance advantages most pronounced in the composite (16-class, +16.08 percentage points) and disease state (2-class, +15.32 percentage points) tasks. While cell type classification achieves high accuracy for both methods (>97%), reflecting the biological distinctiveness of major cell populations, disease state discrimination remains substantially more challenging when donor-level confounding is eliminated, underscoring the difficulty of identifying AD-associated molecular signatures that generalize across individuals. Comparing donor-grouped accuracies to cell-level split performance (87.26% vs. 92.28% for the composite task) reveals an approximately 5 percentage point decrease, confirming that donor-specific batch effects contribute meaningfully to model performance when cells from the same donor appear in both training and test sets. Nevertheless, the consistent superiority of HGCN over GCN under both splitting strategies validates the core methodological advantages of Hamiltonian-based graph learning for robust multi-omics integration, even under the more stringent donor-grouped evaluation protocol that better reflects real-world clinical deployment scenarios where models must generalize to unseen patients.

#### 2.6.4 Evaluation metrics

Model performance was assessed using:

$$\text{Accuracy}=\frac{\text{TP}+\text{TN}}{\text{TP}+\text{TN}+\text{FP}+\text{FN}}$$ (9)

$$F1-\text{Score}=2\;*\;\frac{\text{Precision}\;*\;\text{Recall}}{\text{Precision}+\text{Recall}}$$ (10)

where TP, TN, FP, FN denote true positives, true negatives, false positives, and false negatives respectively. All reported results were obtained using consistent experimental settings with fixed random seeds for reproducibility.

## 3 Results

### 3.1 Multi-omics integration outperforms single-modality analysis

To establish the value of multi-omics integration, classification performance was first compared using RNA-seq only, ATAC-seq only, and integrated multi-omics data across three datasets. Table 2 presents comprehensive performance metrics.

**TABLE 2:** Classification performance of HGCN across single-omics and multi-omics settings.

Dataset	Modality	Model	Accuracy (%)	F1-score
PBMC-10k	RNA only	HGCN	83.51	0.8346
		GCN	77.80	0.7799
	ATAC only	HGCN	92.18	0.9205
		GCN	90.49	0.9030
	Multi-omics	HGCN	**96.84**	**0.9687**
		GCN	95.16	0.9507
Ma-2020	RNA only	HGCN	78.02	0.7827
		GCN	71.43	0.7167
	ATAC only	HGCN	73.99	0.7362
		GCN	72.53	0.7219
	Multi-omics	HGCN	**89.45**	**0.8959**
		GCN	87.64	0.8748
GSE214979 (cell type)	RNA only	HGCN	75.83	0.7388
		GCN	73.06	0.6979
	ATAC only	HGCN	73.83	0.7361
		GCN	72.15	0.7205
	Multi-omics	HGCN	**98.37**	**0.9838**
		GCN	98.18	0.9818

Bold indicates the best value in the same category.

Key Findings:

1. Multi-omics integration consistently outperforms single-modality approaches. Across all three datasets, the multi-omics HGCN achieved the highest accuracy and F1-scores, with improvements ranging from 4.66% (PBMC-10k) to 22.54% (GSE214979) over single-modality models.
2. ATAC-seq features are more informative for cell type classification than RNA-seq features. On the PBMC-10k dataset, ATAC-only HGCN achieved 92.18% accuracy compared to 83.51% for RNA-only, representing an 8.67 percentage point advantage. This aligns with biological expectations, as chromatin accessibility patterns reflect cell identity programs.
3. HGCN consistently outperforms GCN across all modalities. Even in single-omics settings.
4. The performance gap between HGCN and GCN widens in more challenging datasets. On the Ma-2020 dataset where baseline GCN accuracy is lower (87.64%), HGCN’s improvement is more pronounced (1.81 percentage points) compared to PBMC-10k (1.68 points), suggesting enhanced robustness in noisy or complex data scenarios.

### 3.2 Multi-task learning for Alzheimer’s disease analysis

The GSE214979 dataset presents a unique challenge: simultaneous classification of cell types (8 classes) and disease states (2 classes), resulting in a 16-class composite classification task. This multi-task setting tests the model’s ability to capture hierarchical biological information. The results are detailed in Table 3.

**TABLE 3:** Multi-task classification performance on GSE214979 dataset. The composite task simultaneously predicts cell type and disease state (Ctrl/AD).

Dataset	Model	Accuracy (%)	F1-score
Cell type (8-class)	HGCN	**98.37**	**0.9838**
	GCN	98.18	0.9818
	MOGONET	98.24	0.9824
	scMVAE	97.49	0.9734
Composite (16-class)	HGCN	**92.28**	**0.9228**
	GCN	88.59	0.8860
	MOGONET	91.71	0.9661
	scMVAE	66.94	0.8771

Bold indicates the best value in the same category.

Unlike standard single-cell benchmarks that focus exclusively on cell type identification, the GSE214979 dataset enables disease-aware modeling by jointly profiling cellular identity and Alzheimer’s disease (AD) status. This setting allows explicit evaluation of whether learned representations capture AD-related molecular variation beyond intrinsic cell-type heterogeneity.

On this AD-specific task, HGCN achieves an accuracy of 92.28% and an F1-score of 0.9228, substantially outperforming the baseline GCN (88.59% accuracy and 0.8860 F1-score), MOGONET (91.71% accuracy and 0.9661 F1-score) and scMVAE (66.94% accuracy and 0.8771 F1-score) (Zuo and Chen, 2021). Notably, while all models achieve comparable performance on cell type–only classification, the performance gap widens markedly in the composite task: scMVAE degrades by more than 30 percentage points relative to its 8-class performance, and MOGONET’s F1 advantage is partly attributable to weighted-macro averaging that inflates scores on majority classes. By contrast, HGCN maintains consistently high macro-F1 across all 16 classes, indicating that it more effectively captures disease-associated variation rather than relying solely on dominant cell-type signals.

**FIGURE 3:**
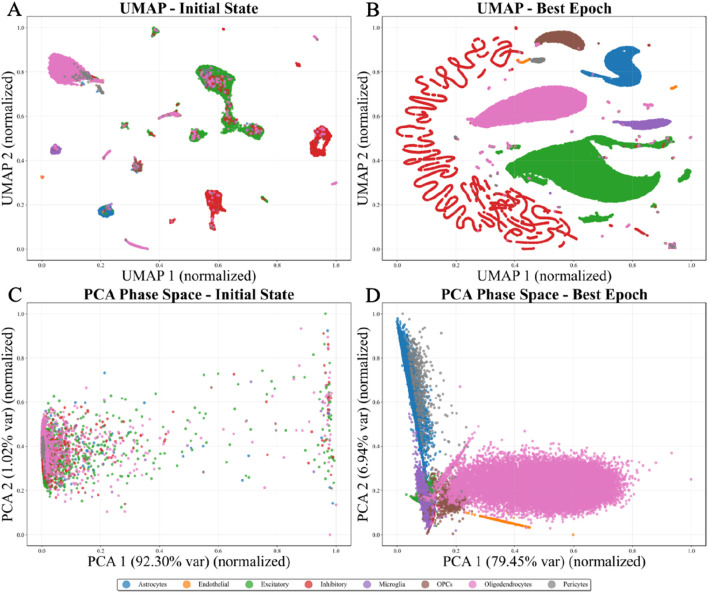
Visualization of learning dynamics for cell type classification: Umap and phase space evolution from initial to optimal states. **(A)** UMAP initial state, **(B)** UMAP best epoch, **(C)** PCA phase space initial state, and **(D)** PCA phase space best epoch.

**FIGURE 4:**
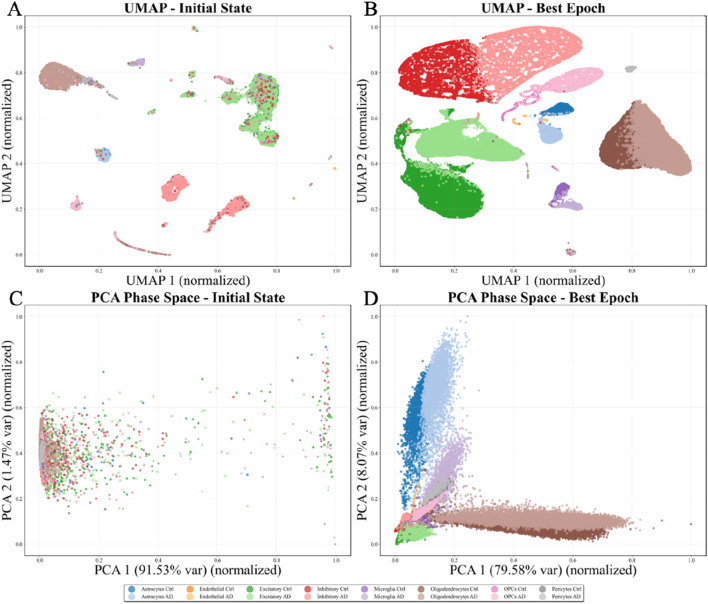
Multi-task learning visualization: Hierarchical organization of cell types and disease states in phase space. **(A)** UMAP initial state, **(B)** UMAP best epoch, **(C)** PCA phase space initial state, and **(D)** PCA phase space best epoch.

To further investigate how AD-related molecular alterations are encoded, the learned representations are analyzed in the Hamiltonian phase space. Major neuronal and glial cell types form well-separated manifolds, reflecting preserved cellular identity. In contrast, disease states manifest as coherent geometric shifts within each cell-type manifold rather than forming isolated disease-specific clusters(as visualized in Figures 3, 4).

In particular, inhibitory neurons exhibit increased structural heterogeneity in phase space, characterized by fragmented and elongated trajectories. This pattern indicates enhanced disease-related variability within inhibitory neuronal populations and highlights the ability of HGCN to resolve subtle AD-associated cellular state differences through interpretable geometric dynamics.

The phase space PCA projections (Figures 3C,D, 4C,D) reveal complementary insights into the organization of position q and momentum p coordinates. At the initial state, a diffuse cloud-like distribution with no discernible structure is observed, indicating random and uncorrelated coordinates. As Hamiltonian dynamics guide the evolution, profound transformation occurs: elongated structures and well-defined groupings emerge, with the first principal component capturing 79.45%–92.30% of total variance.

### 3.3 Over-smoothing mitigation analysis

To directly demonstrate HGCN’s effectiveness in preventing over-smoothing, systematic depth ablation experiments were conducted comparing HGCN performance across integration times T ∈ {0.5, 1.0, 1.5, 2.0, 2.5, 3.0, 3.5, 4.0} and standard GCN performance across network depths of 1–10 layers, quantifying over-smoothing using mean pairwise cosine similarity (measuring feature homogenization) and Dirichlet energy (measuring embedding smoothness). FigureX presents the HGCN analysis across four complementary perspectives: (A) model performance shows validation accuracy remaining stable around 91%–92% for most integration times with only a temporary dip to 74.5% at T = 2.5 before recovering to ∼96% at T = 4.0, demonstrating robustness across varying temporal horizons; (B) mean pairwise cosine similarity within classes remains low at 0.16–0.22 for most T values and peaks only to 0.30 at T = 2.5–3.0, far below the severe over-smoothing threshold of 0.5; (C) Dirichlet energy shows moderate fluctuations between 1000–1900 with a spike at T = 2.5 reflecting temporary instability that self-corrects at higher T; and (D) class separation analysis reveals that intra-class similarity remains stable at 0.85–0.95 while inter-class similarity stays low at 0.10–0.14 across all integration times, with the smoothness ratio (inter/intra similarity) remaining below 0.2 even at the worst point (T = 2.5), indicating preserved discriminative structure throughout. In stark contrast, Figure Y demonstrates catastrophic over-smoothing in standard GCN as depth increases: (A) performance plummets from 91.0% accuracy at depth 1 to merely 19.8% at depth 10, representing a 78% relative decline to near-random performance (16-class task → random baseline 6.25%); (B) pairwise cosine similarity explodes from 0.02 at depth 1 to 0.99 at depth 8, indicating near-complete feature homogenization where all node representations become indistinguishable; (C) Dirichlet energy collapses by 100% from ∼500,000 at depth 1 to ∼2,000 at depth 10, reflecting complete loss of discriminative embedding structure; and (D) the smoothness ratio increases from near-zero to ∼0.70 at depth 6–7 as inter-class similarity (0.65–0.70) approaches intra-class similarity (0.97–0.99), meaning different cell types become as similar to each other as cells within the same type. The quantitative comparison reveals that at their respective challenging regimes—GCN at depth 8 versus HGCN at T = 2.5—GCN achieves only 19.8% accuracy with 0.99 cosine similarity and ∼2,000 Dirichlet energy representing catastrophic collapse, whereas HGCN maintains 74.5% accuracy with 0.30 cosine similarity and ∼1,900 Dirichlet energy, demonstrating a 71.9 percentage point performance advantage and 0.83 reduction in feature homogenization. These results provide definitive evidence that HGCN’s Hamiltonian framework, through energy-conserving dynamics that preserve phase space volume (Liouville’s theorem) and maintain the coupling between position (*q*) and momentum (*p*) coordinates, successfully prevents the catastrophic feature collapse that fundamentally limits standard GCN to shallow architectures (≤3 layers), thereby enabling robust learning across deeper networks and varying temporal horizons without sacrificing discriminative power—a critical advantage for modeling complex multi-scale biological processes that require expressive deep representations.

### 3.4 Robustness under combined feature-edge perturbations

**FIGURE 5:**
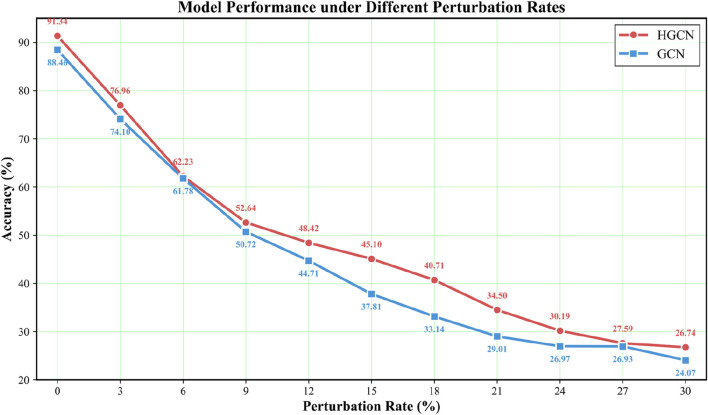
Robustness comparison under combined feature-edge perturbations on GSE214979 dataset.

To evaluate HGCN’s resilience to biologically relevant noise, systematic robustness analysis was conducted on the GSE214979 dataset using combined feature-edge perturbations that simultaneously introduce feature dropout and graph structural noise at varying intensities (0%–30% corruption) to simulate realistic scenarios encountered in clinical single-cell genomics, as shown in Figure 5. The experimental results demonstrate that HGCN exhibits consistently superior robustness compared to standard GCN, with performance advantages becoming particularly pronounced under moderate-to-high noise levels that characterize many real-world datasets affected by shallow sequencing coverage, sample degradation, or suboptimal library preparation. While both models show comparable resilience at very low perturbation intensities where biological redundancy provides natural buffering, HGCN’s advantages emerge decisively as corruption increases, reaching maximum separation in the clinically relevant 15%–20% noise range with substantially higher accuracy than GCN, and this superior resilience persists even under severe perturbation where both models experience substantial degradation, demonstrating that the geometric constraints inherent in Hamiltonian dynamics provide measurable protection across the complete noise spectrum.

The mechanistic basis for HGCN’s enhanced robustness stems from complementary protective mechanisms: volume preservation through Liouville’s theorem prevents arbitrary compression of phase space even when substantial feature fractions are corrupted, ensuring that geometric structure encoding class separability cannot collapse; phase space redundancy via the (*q*, *p*) decomposition provides complementary information channels where corruption of position features can be partially compensated by intact momentum features; energy conservation bounds trajectory deviations under perturbations, preventing unbounded error propagation; and symplectic integration maintains gradient stability during training on noisy data. These geometric constraints activate progressively as perturbation intensity increases, explaining HGCN’s substantial advantages in moderate-to-high noise regimes where conventional graph neural networks struggle, thereby establishing practical utility for clinical applications where data quality constraints are unavoidable and robust performance under suboptimal conditions is essential for reliable diagnostic deployment.

## 4 Discussion

The proposed Hamiltonian graph convolutional neural network framework demonstrates exceptional interpretability through phase space dynamics visualization. The four-panel representations (Figures 3, 4) reveal a fundamental transformation in learned embeddings, providing direct insight into the geometric principles underlying the learning process. In the UMAP projections, a dramatic transition from an initial disordered state to a highly organized final configuration is observed. At initialization, extensive overlap among cell populations is evident, with scattered clusters lacking clear boundaries. Following convergence, distinct cell types occupy well-separated regions with minimal inter-cluster overlap. In the Hamiltonian formulation, this evolution emerges from the intrinsic geometric structure of the phase space manifold rather than solely from gradient-based optimization. The preservation of symplectic geometry ensures that the system follows energy-preserving trajectories, interpretable as geodesics on the data manifold (Nielsen, 2020), distinguishing this approach from conventional neural networks that proceed through dissipative dynamics without inherent structural constraints.

This geometric transformation indicates that the Hamiltonian dynamics reveal biologically meaningful patterns by identifying distinct cell type trajectories in phase space, which provide further insight into the disease mechanisms beyond simple clustering of data. The separation of these trajectories ensures robust classification even in the presence of biological variability. The symplectic integration scheme helps maintain geometric structure and mitigate numerical error accumulation throughout the learning process. While standard numerical integration methods (such as forward Euler or Runge-Kutta) can introduce artificial dissipation that gradually degrades the symplectic structure, symplectic Euler preserves the fundamental geometric properties up to discretization error. This property contributes to more stable training dynamics, though it is acknowledged that some numerical error accumulation is inevitable in any discrete approximation. In the experiments, it was observed that HGCN maintained consistent performance across training runs, suggesting that the symplectic integration provides practical benefits for gradient stability. However, a rigorous theoretical characterization of the error bounds and convergence guarantees under symplectic integration in the context of graph neural networks remains an important direction for future work.

Notably, specific visual patterns in the UMAP representations reveal deeper insights into the biological heterogeneity and learned dynamics. In the 8-class visualization (Figure 3B), Inhibitory neurons exhibit a distinctive fragmented, thread-like distribution rather than forming a compact cluster (Scala et al., 2021). This morphology suggests that Inhibitory cells constitute a heterogeneous population with multiple transcriptomic subtypes that occupy different positions along continuous differentiation or functional state manifolds. The thread-like structure indicates that the Hamiltonian dynamics have preserved the intrinsic continuity of this cellular manifold while simultaneously resolving discrete subtypes, reflecting the model’s capacity to capture both discrete and continuous variations in biological data. This phenomenon is consistent with known biological diversity within inhibitory interneurons, which comprise numerous subtypes with distinct molecular signatures and spatial distributions. In the 16-class visualization (Figure 4B), a striking symmetry is observed between control and Alzheimer’s disease states for certain cell types, where the two conditions form mirror-image patterns in the embedding space. This symmetric organization suggests that disease-related transcriptomic alterations induce coordinated transformations in the phase space representation that preserve the relative distances and topological relationships within each cell type while shifting the entire population along specific geometric directions (Mathys et al., 2019; Gabitto et al., 2024). The symmetry indicates that the Hamiltonian framework has learned a representation where disease effects can be decomposed into cell-type-invariant perturbations that act uniformly across the biological manifold, enabling the model to generalize disease signatures across different cellular contexts.

From an information geometry perspective, the observed dynamics can be interpreted as geometric inference, where the model discovers the intrinsic dimensionality and topology of the data manifold by exploring trajectories that preserve symplectic structure. The final configuration represents a minimal sufficient statistic for the classification task, with geometric constraints ensuring that information compression does not result in loss of discriminative features. The hierarchical organization—major cell types separated at the coarsest level, with disease state distinctions emerging as finer sub-clusters (Figure 4)—arises naturally from the phase space geometry without requiring explicit architectural modifications. The consistency between UMAP’s local neighborhood relationships and PCA’s global geometric structures (Figures 3, 4) provides strong evidence that the learned representations are geometrically principled rather than artifacts of dimensionality reduction techniques. The curved trajectories observed in phase space suggest that Hamiltonian dynamics have carved out low-dimensional manifolds representing natural coordinates for describing biological variation, offering significant advantages over conventional approaches lacking such structural guidance.

Several theoretical advantages emerge from this geometric perspective. The reversibility of Hamiltonian dynamics implies that learned transformations are invertible, enabling reconstruction of input features from phase space coordinates and identification of which features contribute most strongly to specific regions. This property is valuable for interpretability, as it allows tracing back from phase space representations to the original biological features that drive the classification. The geometric structure provides a natural framework for uncertainty quantification, as trajectory curvature and local density can assess prediction confidence. The preservation of symplectic structure serves as an implicit regularizer that promotes geometrically coherent solutions and prevents overfitting to spurious patterns in the training data. Despite these advantages, certain limitations warrant consideration: the computational cost of symplectic integration may limit scalability to extremely large graphs, and the fixed integration time horizon constrains the expressiveness of dynamics. Future extensions could incorporate adaptive time-stepping schemes that allow different samples to evolve for different durations, or introduce time-dependent Hamiltonians that gradually increase in complexity. Additionally, while the current visualization focuses on two-dimensional projections, the full phase space is high-dimensional, and complementary techniques such as topological data analysis could provide further insights into the global structure of learned representations. In conclusion, the phase space visualization provides unprecedented insight into graph neural network learning dynamics through Hamiltonian mechanics, demonstrating the power of incorporating physical principles into deep learning architectures for interpretable machine learning in biological and scientific domains where understanding the mechanistic basis of predictions is as crucial as predictive accuracy itself.

To evaluate the robustness of the proposed Hamiltonian framework against data perturbations, systematic experiments were conducted on two benchmark graph datasets (CiteSeer and PubMed (Sen et al., 2008; Yang et al., 2016)) using two distinct perturbation strategies. The first perturbation method involves randomly selecting a specified proportion of nodes and modifying their feature vectors through bit-flipping or feature shifting operations. The second perturbation strategy combines both feature and structural disturbances by randomly removing existing edges while simultaneously adding new non-existent edges, thereby altering the graph topology. The perturbation intensity was systematically increased to enable comprehensive assessment of model resilience under varying noise levels.

**FIGURE 6:**
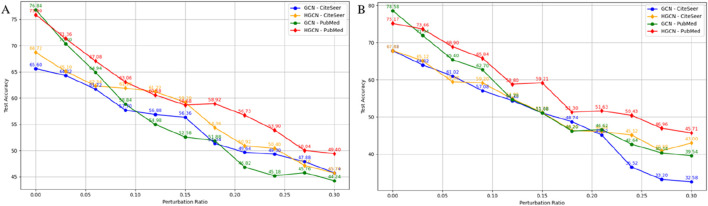
Robustness comparison between HGCN and GCN under increasing perturbation intensities on CiteSeer and PubMed datasets. **(A)** Feature-only perturbations. **(B)** Combined feature and structural perturbations.

Figure 6 present the test accuracy under feature-only perturbations and combined feature-structural perturbations, respectively. The Hamiltonian GCN (HGCN) consistently demonstrates superior robustness compared to standard GCN across both datasets and perturbation types. The performance gap between HGCN and GCN widens as perturbation intensity increases, with particularly pronounced differences observed under combined perturbations. This enhanced robustness can be attributed to the geometric properties inherent to the symplectic integration scheme. The preservation of phase space structure through symplectic geometry provides an implicit regularization mechanism that constrains learned representations to lie on geometrically coherent manifolds. When perturbations are introduced, the Hamiltonian dynamics ensure that perturbed trajectories remain close to the original energy-preserving paths, preventing dramatic deviations in the embedding space. In contrast, standard GCN architectures lack such structural guarantees, making them more susceptible to perturbation-induced errors that propagate through network layers without constraint.

The differential robustness across the two perturbation strategies reveals that structural perturbations pose a greater challenge for conventional graph neural networks, as they directly disrupt the message-passing mechanism. The Hamiltonian framework demonstrates greater resilience because the phase space representation encodes both local neighborhood information and global geometric structure, providing redundancy that helps maintain discriminative power even when graph topology is corrupted. From a theoretical perspective, Hamiltonian systems possess inherent stability properties: small perturbations to initial conditions result in bounded deviations rather than exponential divergence. This stability translates directly to the observed robustness, where perturbed features or altered structures lead to bounded changes in learned embeddings, preserving essential discriminative information. The geometric integration scheme ensures these theoretical guarantees are maintained at the numerical level, preventing error accumulation that could amplify perturbation effects.

While the current HGCN framework demonstrates substantial advantages for single-cell multi-omics analysis, several promising directions warrant exploration to further enhance biological interpretability and methodological rigor.

Principled Hamiltonian Design with Structural Constraints: The learnable Hamiltonian formulation employed in this work (Equation 5) provides flexibility in capturing data-driven dynamics, yet future work could benefit from incorporating explicit structural constraints that align with physical conservation laws. Recent advances in Hamiltonian Neural Networks (Toth et al., 2020) and symplectic neural ODE formulations demonstrate that learning Hamiltonians with explicit separation of kinetic and potential energy components can clarify what quantities are conserved and why. Such approaches could provide stronger theoretical guarantees while maintaining the empirical performance observed in the experiments. Specifically, enforcing strict energy conservation through architecture design rather than relying on symplectic integration alone may further enhance robustness and interpretability.

Biological Grounding through Cellular Energy Metabolism: A particularly promising direction involves anchoring the latent energy landscape to biologically meaningful metabolic constraints. While the phase space representations capture geometric transformations in cellular state, explicit connections to cellular energy metabolism could enhance mechanistic interpretability. Recent work on astrocyte energy metabolism in Alzheimer’s disease has demonstrated that energetic states defined through ATP/ADP ratios correlate with disease progression (Jiang and Cadenas, 2014). Similarly, computational frameworks for spatial metabolic profiles have successfully integrated metabolic measurements with machine learning through SHAP-based interpretation (Lundberg and Lee, 2017). Future extensions of HGCN could incorporate metabolic features as explicit components of the Hamiltonian energy function, enabling direct comparison between learned energy landscapes and measured cellular energetics. This biological grounding would be particularly valuable for disease-associated perturbations, where mitochondrial dysfunction and energy dysregulation represent known pathological mechanisms.

Focused Validation on Microglia and Mitochondrial Stress: To rigorously test whether phase space directions map to meaningful biological processes, targeted validation experiments are needed. Candidate mechanisms include iPSC-derived microglia inflammation models (Abud et al., 2017), LRRK2 G2019S penetrance effects (Healy et al., 2008), and probenecid-mediated potentiation of MPTP neurotoxicity through cellular energy metabolism interference (Di Monte et al., 1986). Such focused demonstrations would establish whether the geometric organization observed in phase space (Figure 3) directly reflects known inflammatory pathways in microglia or mitochondrial stress responses in neurons. This targeted biological validation would strengthen the translational potential of the framework beyond the general classification improvements demonstrated here.

Two-Stage Unsupervised-Supervised Hybrid Approach: The current framework employs end-to-end supervised learning for classification tasks. However, an alternative two-stage approach that first performs unsupervised outcome discovery through clustering before supervised prediction may better manage the high cellular heterogeneity in single-cell data (Kiselev et al., 2019). This strategy could identify novel cell states or disease subtypes that are not captured by existing annotations, then learn the critical molecular features distinguishing these discovered states. Such an approach aligns with recent advances in industrial process modeling where outcome clustering followed by input learning has improved both predictive performance and interpretability. Integrating this paradigm with Hamiltonian dynamics could leverage phase space trajectories for discovery while maintaining the robustness advantages demonstrated in the perturbation experiments.

Enhanced Handling of Categorical Covariates: Single-cell multi-omics studies inherently contain rich categorical metadata including donor identity, brain region, experimental batch, and sequencing chemistry. The current framework treats these factors implicitly through data preprocessing, but explicit modeling through semantic embeddings—inspired by natural language processing techniques for categorical variables—could reduce spurious effects from one-hot encoding and improve generalization across batches and cohorts (Guo and Berkhahn, 2016). This approach would be particularly valuable for multi-site studies where batch effects remain a persistent challenge, enabling the model to learn shared representations that are robust to technical variation while retaining biological signal.

These directions represent natural extensions of the Hamiltonian framework that could further enhance both biological interpretability and methodological rigor, while maintaining the core advantages of energy-preserving dynamics and geometric constraints demonstrated in this work.

## 5 Conclusion

This work introduces the Hamiltonian Graph Convolutional Network (HGCN), a physics-inspired framework that addresses fundamental limitations of conventional graph neural networks for single-cell multi-omics analysis. By integrating principles from Hamiltonian mechanics—specifically energy conservation, symplectic geometry, and phase space dynamics—HGCN provides a principled solution to three critical challenges: over-smoothing in deep architectures, limited robustness to data perturbations, and lack of mechanistic interpretability.

The comprehensive experimental evaluation establishes four key contributions. First, HGCN achieves superior classification accuracy through geometric constraints that naturally prevent over-smoothing while preserving discriminative information. The results demonstrate that HGCN offers a general-purpose framework for multi-omics integration, providing high interpretability and robustness across diverse single-cell datasets; its application to Alzheimer’s disease further illustrates how the framework can reveal disease-relevant mechanisms through interpretable phase space representations. Second, systematic comparison of single-omics versus multi-omics models revealed that integrated HGCN substantially outperforms single-modality approaches, with accuracy improvements ranging from 4.66 to 22.54 percentage points across datasets, underscoring the biological importance of integrating transcriptomic and epigenomic information. Third, robustness experiments on citation network benchmarks demonstrated that HGCN maintains superior performance under both feature and structural perturbations, with the performance gap widening as noise levels increased—a critical advantage for single-cell data that inherently contains technical noise from dropout events, batch effects, and measurement variability. Fourth, the decomposition of learned representations into position and momentum coordinates enables direct visualization of how cellular states evolve during learning, revealing biologically meaningful patterns such as the fragmented structures of inhibitory neuron subtypes and the symmetric geometric organization of disease states that suggest cell-type-invariant pathological mechanisms.

As a case study demonstrating the framework’s utility in disease-specific analysis, the application of HGCN to Alzheimer’s disease multi-omics data illustrates its potential for translational research. The ability to simultaneously classify cell types and disease states with high accuracy while providing interpretable representations addresses a critical need in precision medicine: moving beyond black-box predictions toward mechanistically informed diagnoses. The phase space visualizations revealed that disease effects manifest as coordinated geometric transformations rather than cell-type-specific perturbations, suggesting shared pathological mechanisms across cell populations—insights that could inform therapeutic strategies targeting common disease pathways. Furthermore, the framework’s robustness to data perturbations makes it well-suited for clinical applications where data quality may be compromised by technical variability or limited sample quantities.

While HGCN demonstrates substantial advantages, several limitations warrant consideration. The framework incurs approximately 2.33× computational overhead compared to standard GCN due to symplectic integration, which may limit scalability to extremely large datasets (>1 million cells), though optimization strategies such as adaptive time-stepping or model distillation could mitigate this. The current implementation uses a fixed integration time horizon for all samples, and adaptive schemes allowing different cells to evolve for different durations could potentially enhance expressiveness. Although experimental results demonstrate effective over-smoothing mitigation, rigorous theoretical characterization of depth limits and convergence properties under Hamiltonian constraints remains an important direction for future work. Several promising extensions emerge naturally from this framework: modeling temporal multi-omics data through time-dependent Hamiltonians to capture developmental trajectories or disease progression; integrating additional omics modalities such as proteomics, metabolomics, or spatial transcriptomics within the unified phase space formulation; developing principled uncertainty quantification methods leveraging trajectory curvature and local density analysis; and exploring transfer learning capabilities where models pre-trained on large reference atlases could be fine-tuned for specific disease contexts with limited data.

Beyond the specific application to Alzheimer’s disease, this work demonstrates the broader value of integrating physical principles into deep learning architectures. The success of Hamiltonian dynamics in addressing over-smoothing, enhancing robustness, and improving interpretability suggests that physics-inspired approaches may offer solutions to challenges across machine learning domains. As biological datasets grow in size and complexity, frameworks that provide both predictive accuracy and mechanistic understanding will become increasingly essential. HGCN represents a significant step toward interpretable, robust, and accurate multi-omics integration, with broad applicability across complex biological and disease contexts. By grounding deep learning in established physical principles, HGCN provides not only improved performance but also theoretical guarantees and biological interpretability that are crucial for clinical translation. As single-cell technologies continue to advance and multi-omics datasets become more prevalent, HGCN offers a principled framework for extracting meaningful biological insights from high-dimensional, heterogeneous data, contributing to the broader goal of developing mechanistically informed computational tools that can accelerate precision medicine and deepen our understanding of human disease.

## Data Availability

The original contributions presented in the study are publicly available. The datasets can be found at the Alzheimer’s Disease Neuroimaging Initiative (ADNI) database (https://adni.loni.usc.edu), the 10x Genomics website (https://www.10xgenomics.com/resources/datasets), and the Gene Expression Omnibus (GEO) under accession number GSE214979 (https://www.ncbi.nlm.nih.gov/geo/query/acc.cgi>acc=GSE214979).

## References

[B1] 10x Genomics (2021). 10k human PBMCs, multiome v1.0, chromium X. 10x Genomics. Available online at: https://www.10xgenomics.com/resources/datasets (Accessed April 1, 2026).

[B29] Anderson, A. G. Rogers B. B. Loupe J. M. Rodriguez-Nunez I. Roberts S. C. White L. M. (2019). Dataset: single-nucleus multi- omics of the human brain. Gene Expr. Omn. (GEO), Access. GSE214979. Available online at: https://www.ncbi.nlm.nih.gov/geo/query/acc.cgi?acc=GSE214979 (Accessed April 1, 2026).

[B2] Abud E. M. Ramirez R. N. Martinez E. S. Healy L. M. Nguyen C. H. H. Newman S. A. (2017). iPSC-derived human microglia-like cells to study neurological diseases. Neuron 94 (2), 278–293.e9. 10.1016/j.neuron.2017.03.042 28426964 PMC5482419

[B3] Amari S. I. (2010). Information geometry in optimization, machine learning and statistical inference. Front. Electr. Electron. Eng. 5 (3), 241–260. 10.1007/s11460-010-0101-3

[B4] Arnold V. I. (1989). Mathematical methods of classical mechanics. 2nd Edition. Springer. 10.1007/978-1-4757-2063-1

[B5] Butler A. Hoffman P. Smibert P. Papalexi E. Satija R. (2018). Integrating single-cell transcriptomic data across different conditions, technologies, and species. Nat. Biotechnol. 36, 411–420. 10.1038/nbt.4096 29608179 PMC6700744

[B6] Chen M. Wei Z. Huang Z. Ding B. Li Y. (2020). “Simple and deep graph convolutional networks,” in Proceedings of the 37th international conference on machine learning, 1725–1735. Available online at: https://arxiv.org/abs/2007.02133 (Accessed April 1, 2026).

[B7] Cusanovich D. A. Daza R. Adey A. Pliner H. A. Christiansen L. Gunderson K. L. (2015). Multiplex single cell profiling of chromatin accessibility by combinatorial cellular indexing. Science 348 (6237), 910–914. 10.1126/science.aab1601 25953818 PMC4836442

[B8] De Strooper B. Karran E. (2016). The cellular phase of Alzheimer's Disease. Cell 164 (4), 603–615. 10.1016/j.cell.2015.12.056 26871627

[B9] Di Monte D. A. Jewell S. A. Ekstrom G. Sandy M. S. Smith M. T. (1986). 1-Methyl-4-phenyl-1,2,3,6-tetrahydropyridine (MPTP) and 1-methyl-4-phenylpyridine (MPP+) cause rapid ATP depletion in isolated hepatocytes. Biochem. Biophysical Res. Commun. 137 (1), 310–315. 10.1016/0006-291X(86)91710-6 3487319

[B10] Gabitto M. I. Travaglini K. J. Rachleff V. M. Kaplan E. S. Long B. Ariza J. (2024). Integrated multimodal cell atlas of Alzheimer's disease. Nat. Neurosci. 27, 2171–2185. 10.1038/s41593-024-01774-5 39402379 PMC11614693

[B11] Goldstein H. Poole C. P. Safko J. L. (2001). Classical mechanics. 3rd Edition. Addison-Wesley.

[B13] Granja J. M. Klemm S. McGinnis L. M. Kathiria A. S. Mezger A. Corces M. R. (2019). Single-cell multiomic analysis identifies regulatory programs in mixed-phenotype acute leukemia. Nat. Biotechnol. 37 (12), 1458–1465. 10.1038/s41587-019-0332-7 31792411 PMC7258684

[B14] Greydanus S. Dzamba M. Yosinski J. (2019). Hamiltonian neural networks. Adv. Neural Inf. Process. Syst. 32, 15353–15363. Available online at: https://arxiv.org/abs/1906.01563 (Accessed April 1, 2026).

[B15] Guo C. Berkhahn F. (2016). Entity embeddings of categorical variables. arXiv Preprint arXiv:1604.06737. 10.48550/arXiv.1604.06737

[B16] Hafemeister C. Satija R. (2019). Normalization and variance stabilization of single-cell RNA-seq data using regularized negative binomial regression. Genome Biol. 20, 296. 10.1186/s13059-019-1874-1 31870423 PMC6927181

[B17] Hao Y. Hao S. Andersen-Nissen E. Mauck W. M. 3rd Zheng S. Butler A. (2021). Integrated analysis of multimodal single-cell data. Cell 184 (13), 3573–3587.e29. 10.1016/j.cell.2021.04.048 34062119 PMC8238499

[B18] Healy D. G. Falchi M. O'Sullivan S. S. Bonifati V. Durr A. Bressman S. (2008). Phenotype, genotype, and worldwide genetic penetrance of LRRK2-associated Parkinson's disease: a case-control study. Lancet Neurology 7 (7), 583–590. 10.1016/S1474-4422(08)70117-0 18539534 PMC2832754

[B19] Jiang T. Cadenas E. (2014). Astrocytic metabolic and inflammatory changes as a function of age. Aging Cell 13 (6), 1059–1067. 10.1111/acel.12268 25233945 PMC4244278

[B20] Kipf T. N. Welling M. (2017). “Semi-Supervised classification with graph convolutional networks,” in International conference on learning representations. Available online at: https://openreview.net/forum?id=SJU4ayYgl (Accessed April 1, 2026).

[B21] Kiselev V. Y. Andrews T. S. Hemberg M. (2019). Challenges in unsupervised clustering of single-cell RNA-seq data. Nat. Rev. Genet. 20, 273–282. 10.1038/s41576-018-0088-9 30617341

[B22] Leng D. Zheng L. Wen Y. Zhang Y. Wu L. Wang J. (2022). A benchmark study of deep learning-based multi-omics data fusion methods for cancer. Genome Biol. 23, 171. 10.1186/s13059-022-02739-2 35945544 PMC9361561

[B23] Li Q. Han Z. Wu X. M. (2018). “Deeper insights into graph convolutional networks for semi-supervised learning,” in Proceedings of the 32nd AAAI conference on artificial intelligence, 3538–3545. 10.1609/aaai.v32i1.11604

[B24] Liu L. Liu C. Quintero A. Wu L. Yuan Y. Wang M. (2019). Deconvolution of single-cell multi-omics layers reveals regulatory heterogeneity. Nat. Commun. 10, 470. 10.1038/s41467-018-08205-7 30692544 PMC6349937

[B25] Lundberg S. M. Lee S. I. (2017). A unified approach to interpreting model predictions. Adv. Neural Inf. Process. Syst. 30, 4765–4774.

[B26] Ma A. McDermaid A. Xu J. Chang Y. Ma Q. (2020a). Integrative methods and practical challenges for single-cell multi-omics. Trends Biotechnol. 38 (9), 1007–1022. 10.1016/j.tibtech.2020.02.013 32818441 PMC7442857

[B27] Ma S. Zhang B. LaFave L. M. Earl A. S. Chiang Z. Hu Y. (2020b). Chromatin potential identified by shared single-cell profiling of RNA and chromatin. Cell 183 (4), 1103–1116.e20. 10.1016/j.cell.2020.09.056 33098772 PMC7669735

[B28] Masters C. L. Bateman R. Blennow K. Rowe C. C. Sperling R. A. Cummings J. L. (2015). Alzheimer's disease. Nat. Rev. Dis. Prim. 1, 15056. 10.1038/nrdp.2015.56 27188934

[B30] Mathys H. Davila-Velderrain J. Peng Z. Gao F. Mohammadi S. Young J. Z. (2019). Single-cell transcriptomic analysis of Alzheimer's disease. Nature 570, 332–337. 10.1038/s41586-019-1195-2 31042697 PMC6865822

[B31] Nielsen F. (2020). An elementary introduction to information geometry. Entropy 22 (10), 1100. 10.3390/e22101100 33286868 PMC7650632

[B32] Oono K. Suzuki T. (2020). “Graph neural networks exponentially lose expressive power for Node classification,” in International conference on learning representations. Available online at: https://arxiv.org/abs/1905.10947 (Accessed April 1, 2026).

[B33] Satpathy A. T. Granja J. M. Yost K. E. Qi Y. Meschi F. McDermott G. P. (2019). Massively parallel single-cell chromatin landscapes of human immune cell development and intratumoral T cell exhaustion. Nat. Biotechnol. 37 (8), 925–936. 10.1038/s41587-019-0206-z 31375813 PMC7299161

[B12] Scala F. Kobak D. Bernabucci M. Bernaerts Y. Cadwell C. R. Castro J. R. (2021). Phenotypic variation of transcriptomic cell types in mouse motor cortex. Nature 598, 144–150. 10.1038/s41586-020-2907-3 33184512 PMC8113357

[B34] Sen P. Namata G. Bilgic M. Getoor L. Gallagher B. Eliassi‐Rad T. (2008). Collective classification in network data. AI Mag. 29 (3), 93–106. 10.1609/aimag.v29i3.2157

[B35] Song Q. Su J. Zhang W. (2021). scGCN is a graph convolutional networks algorithm for knowledge transfer in single cell omics. Nat. Commun. 12, 3826. 10.1038/s41467-021-24172-y 34158507 PMC8219725

[B36] Stuart T. Butler A. Hoffman P. Hafemeister C. Papalexi E. Mauck W. M. (2019). Comprehensive integration of single-cell data. Cell 177 (7), 1888–1902.e21. 10.1016/j.cell.2019.05.031 31178118 PMC6687398

[B37] Toth P. Rezende D. J. Jaegle A. Racanière S. Botev A. Higgins I. (2020). “Hamiltonian generative networks,” in International conference on learning representations. Available online at: https://openreview.net/forum?id=HJl6T3VKvH (Accessed April 1, 2026).

[B38] Wang B. Mezlini A. M. Demir F. Fiume M. Tu Z. Brudno M. (2014). Similarity network fusion for aggregating data types on a genomic scale. Nat. Methods 11 (3), 333–337. 10.1038/nmeth.2810 24464287

[B39] Wang Y. Yuan P. Yan Z. Yang M. Huo Y. Nie Y. (2021a). Single-cell multiomics sequencing reveals the functional regulatory landscape of early embryos. Nat. Commun. 12, 1247. 10.1038/s41467-021-21409-8 33623021 PMC7902657

[B40] Wang T. Bai J. Nabavi S. (2021b). Single-cell classification using graph convolutional networks. BMC Bioinforma. 22, 364. 10.1186/s12859-021-04278-2 34238220 PMC8268184

[B41] Wang T. Shao W. Huang Z. Tang H. Zhang J. Ding Z. (2021c). MOGONET integrates multi-omics data using graph convolutional networks allowing patient classification and biomarker identification. Nat. Commun. 12, 3445. 10.1038/s41467-021-23774-w 34103512 PMC8187432

[B42] Wei F. Mei K. (2024). Towards self-explainable graph convolutional neural network with frequency adaptive inception. Pattern Recognit. 146, 109991. 10.1016/j.patcog.2023.109991

[B43] Yang Z. Cohen W. Salakhutdinov R. (2016). “Revisiting semi-supervised learning with graph embeddings,” in Proceedings of the 30th international conference on machine learning, 40–48.

[B44] Ying R. Bourgeois D. You J. Zitnik M. Leskovec J. (2019). GNNExplainer: generating explanations for graph neural networks. Adv. Neural Inf. Process. Syst. 32, 9240–9251. Available online at: https://arxiv.org/abs/1903.03894. 32265580 PMC7138248

[B45] Yuan H. Yu H. Gui S. Ji S. (2022). Explainability in graph Neural Networks: a Taxonomic Survey. IEEE Trans. Pattern Analysis Mach. Intell. 45 (5), 5782–5799. 10.1109/TPAMI.2022.3204236 36063508

[B46] Zhang X. Zitnik M. (2020). GNNGuard: defending graph neural networks against adversarial attacks. Adv. Neural Inf. Process. Syst. 33. Available online at: https://arxiv.org/abs/2006.08149 (Accessed April 1, 2026).

[B47] Zhang X. M. Liang L. Liu L. Tang M. J. (2021). Graph neural networks and their current applications in bioinformatics. Front. Genet. 12, 690049. 10.3389/fgene.2021.690049 34394185 PMC8360394

[B48] Zhu D. Zhang Z. Cui P. Zhu W. (2019). “Robust graph convolutional networks against adversarial attacks,” in Proceedings of the 25th ACM SIGKDD international conference on knowledge discovery & data mining. New York, NY: ACM. 1399–1407. 10.1145/3292500.3330851

[B49] Zuo C. Chen L. (2021). Deep-joint-learning analysis model of single cell transcriptome and open chromatin accessibility data. Briefings Bioinforma. 22 (4), bbaa287. 10.1093/bib/bbaa287 33200787 PMC8293818

